# Neurological Conditions Following COVID-19 Vaccinations: Chance or Association?

**DOI:** 10.7759/cureus.21919

**Published:** 2022-02-04

**Authors:** Josaiah Fernandes, Sheneel Jaggernauth, Vanita Ramnarine, Saeed R Mohammed, Chenelle Khan, Avidesh Panday

**Affiliations:** 1 Department of Medicine, The University of the West Indies, St. Augustine Campus, Champ Fleurs, TTO; 2 Department of Medicine, Eric Williams Medical Sciences Complex, Champ Fleurs, TTO

**Keywords:** astra zeneca covid-19 vaccine, pfizer-biontech covid-19 vaccine, pfizer vaccine, neurological side effects, vaccine adverse events, covid 19, vaccine side effects, covid-19 vaccine

## Abstract

Coronavirus disease 2019 (COVID-19) has been labeled a global pandemic with the first reported case of the severe acute respiratory syndrome coronavirus 2 (SARS-CoV-2) occurring in Wuhan, China in December 2019. To combat the alarming, increasing rate of those affected by the virus, vaccine development ensued. As mass vaccination initiatives against COVID-19 ensued, adverse reactions began emerging. This non-consecutive, population-based case series focuses on four vaccine-associated neurological adverse events across the central and peripheral nervous system detailing the diagnosis, treatment and subsequent follow-up management. These four patients presented to public and private hospitals in Trinidad and Tobago with new-onset neurological diseases soon after their first doses of a COVID-19 vaccine: two after the Pfizer-BioNTech vaccine (one case of new-onset seizures and one case of longitudinally extensive transverse myelitis) and two after the ChAdOx1 nCoV-19 vaccine (one case of Guillain-Barre syndrome and one case of meningitis-retention syndrome). The background incidence rates of neurological conditions in the population and the large numbers of persons being vaccinated means that some of these conditions will appear in the post-vaccination window by chance. Hence, establishing causal links is difficult. The close temporal relationship between vaccination and the presenting symptoms, the biological plausibility, and the extensive diagnostic workup to exclude other causes fulfill criteria provided by the World Health Organization for causality assessment of an adverse event following immunization on an individual level. On this basis, it was determined that these adverse events were likely due to the vaccines. However, establishing causal links on a population level requires large epidemiological studies and cannot be done on individual case reports alone. While physicians should be cognizant of even these rare adverse events of vaccines, it should be reiterated that the overall safety profile of vaccines is well established.

## Introduction

Coronavirus disease 2019 (COVID-19), caused by severe acute respiratory syndrome coronavirus 2 (SARS-CoV-2), was declared a global pandemic in March 2020 by the World Health Organization (WHO) [1]. Global initiatives were undertaken to develop vaccines against the pandemic which has affected over 250 million people and claimed over 5 million lives as of October 2021 [2]. As of January 12th, 2022, the World Health Organization currently lists the use of ChAdOx1 nCoV-19 vaccines as safe and effective for persons over 18 while the Pfizer-BioNTech vaccines are suitable for use for persons over the age of five [3]. These two vaccines are the focus of this case report. Worldwide vaccine rollouts against SARS-CoV-2 began and as more persons became inoculated, cases of neurological adverse events following vaccination vaccine were reported [4].

According to the Center for Disease Control and Prevention (CDC), commonly associated side effects of COVID-19 vaccination include but are not limited to: pain and swelling at the injection site, fatigue, headache, nausea, myalgia, chills and fever [5]. However, given the novelty of the COVID-19 vaccines and the limited literature pertaining to potential rarer adverse effects, this case report describes four individually reported cases of neurological symptoms post vaccination. The cases include new-onset seizures following Pfizer-BioNTech, transverse myelitis following Pfizer-BioNTech, meningitis-retention syndrome following the ChAdOx1 nCoV-19 vaccine and Guillain-Barre syndrome following the ChAdOx1 nCoV-19 vaccine. The patients in these cases presented to private and public hospitals in Trinidad and Tobago. This case series should provide useful insight into the presentation of new-onset neurological signs and symptoms, clinical course as well as the efficacy of the treatment plan utilized in these respective cases to potentially aid in the future management of similar post-COVID-19 vaccine symptom presentations.

## Case presentation

Case 1: Temporal association of new-onset seizures and insulin-dependent diabetes mellitus one week after Pfizer-BioNTech vaccine

A 16-year-old previously well male without comorbidities presented to the emergency department with four episodes of witnessed generalized tonic clonic seizures. He was then admitted to the pediatric medical ward. These events occurred one week after he received the first dose of the Pfizer-BioNTech vaccine. One of these events occurred while he was asleep and the others occurred in wakefulness with no aura but with post-ictal phases for all other events. On all occasions, there was a return to baseline function and normal neurological examination.

Further investigations revealed a normal non-contrast magnetic resonance imaging (MRI) Brain (1.5T) as well as an electroencephalogram (EEG) consistent with bitemporal focal slowing with admixed sharp waves, more prominently on the right. Of note, there was intermittent background slowing in the theta range. A lumbar puncture revealed normal opening pressure with 15 white blood cells, normal glucose as well as minimally elevated protein (normal <48mg/dl). A tentative diagnosis of viral encephalitis was made and the patient was initially treated with intravenous phenytoin and subsequently converted to oral maintenance as well as intravenous acyclovir. After a seven-day course of medication with no further seizures or signs of sepsis, the patient was discharged on maintenance phenytoin.

Nine days later, the patient re-presented with further seizure clusters with return to normal baseline and no noticeable disturbance to his premorbid cognitive function or signs of sepsis. The patient underwent another lumbar puncture, which revealed three white blood cells with a markedly elevated glucose at 177mg/dl (serum glucose 351mg/dl) and an increased protein of 79mg/dl. Of note, the elevated blood sugars (>500mg/dl) were not accompanied by ketosis on urine dipstick and without acidosis on blood gas testing. After evaluation by the Endocrinologist, he was diagnosed with new diabetes mellitus and commenced on a basal bolus insulin regimen. 

At this time, owing to poor seizure control, his medications were switched to sodium valproate 400mg twice daily and clonazepam 0.5mg at night. Additionally, his diagnosis was re-examined and given the high cerebrospinal fluid (CSF) protein, in the absence of abnormal cells and negative culture, an infective etiology was deemed less likely and the possibility of an immune-mediated encephalopathy was considered. Specifically, given the encephalopathy as well as new diabetes mellitus, we considered the possibility of anti-glutamic acid decarboxylase (GAD) encephalitis. However, serology related to anti-GAD encephalitis was not available due to our limited resources. 

The patient was treated with a one-week course of dexamethasone due to the unavailability locally of methylprednisolone with an outpatient taper. His basal bolus insulin regimen was adjusted as his new-onset diabetes mellitus had resolved. He followed up in the clinic a month after discharge, with no seizures and normalization of his EEG. A repeat EEG done one month post steroid treatment revealed improvement in the background with minimal right focal slowing and without definite epileptiform activity.

Case 2: Longitudinally extensive transverse myelitis following Pfizer-BioNTech vaccine

A 26-year-old female with no known underlying medical comorbidities presented with a one-day history of acute neck pains as well as rapidly progressing bilateral lower limb weakness and asymmetric left upper limb weakness two days after receiving the first dose of the Pfizer-BioNTech vaccine. Over the ensuing day, she had a rapidly progressive, flaccid paraparesis with increased weakness in the left leg, with a power of ⅕ compared to the right leg which maintained a ⅘ power grading. There was also development of a single episode of urinary and fecal incontinence.

**Figure 1:**
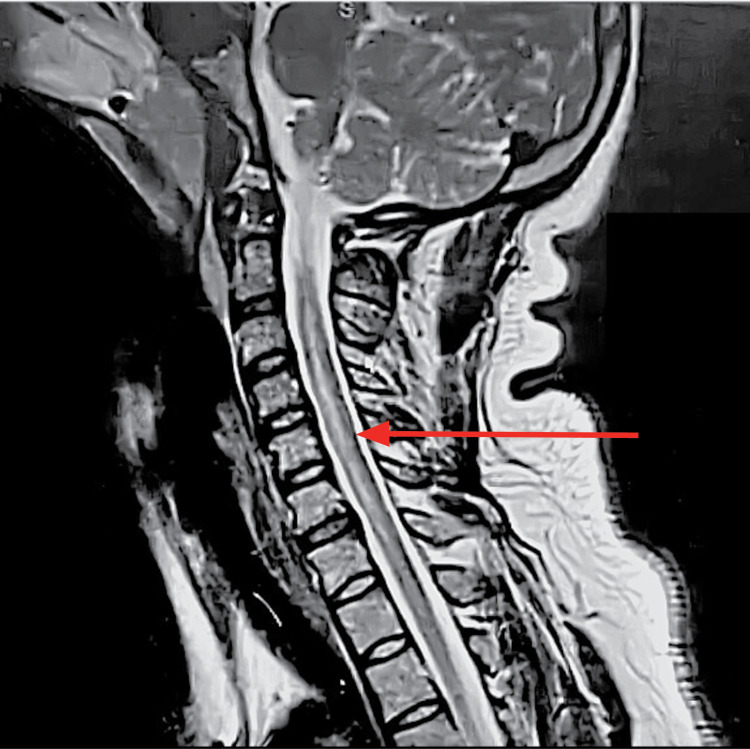
Sagittal, T2-weighted MRI of the cervical spine Red arrow indicating a hyperintense lesion along the central grey matter extending from lower medulla-T1

An MRI of the brain and spine with contrast revealed a longitudinally extensive transverse myelitis (LETM) from the medulla to the T1 level without enhancement (Figure 1). No lesions were noted in the brain. Her CSF analysis revealed normal opening pressure and no abnormal CSF white blood cells and normal protein and glucose levels. Despite repeated attempts, we were unable to ascertain her anti-myelin oligodendrocyte glycoprotein (MOG) and aquaporin IgG4 antibodies. Her antinuclear antibodies (ANA)/dsDNA were negative and her chest x-ray was within normal limits (no mediastinal lymphadenopathy and no parenchymal lung disease).

The patient was commenced on methylprednisolone 1g intravenously for five days and subsequently placed on a tapering course of prednisolone at 1mg/kg/day orally. Given that she fulfilled the clinical and radiological criteria for neuromyelitis optica spectrum disorders (NMOSD), she was treated with a loading dose of rituximab 2g followed by 1g every two weeks intravenously. The follow up plan includes continuing rituximab 1g every six months for the next two years with yearly review of her anti-MOG and aquaporin antibodies.

Over the course of her hospitalization, the patient received physiotherapy which improved movement of the upper limb as well as walking with ambulatory aids. The patient’s bowel and bladder function also returned.

Case 3: Guillain-Barre syndrome following ChAdOx1 nCoV-19 vaccine 

A 50-year-old hypertensive man presented with a one-day history of severe headaches in the temporal region, lower back and umbilical abdominal pain with paresthesias in his hands and feet. Within one day of his admission, he developed bilateral proximal lower limb weakness as well as asymmetric facial weakness resulting in drooling and significant dysarthria. On clinical examination, the patient was lying comfortably with no signs of respiratory distress and vital signs were stable. He was well oriented to time, person and place. His Glasgow Coma Scale (GCS) was 15/15. His gait progressed from normal to a broad based right-sided drift. He had a normal tone and ⅘ power throughout. His reflexes were normal except for his right patellar and Achilles reflexes which were decreased (1+). 

The patient denied any family history, presence of photophobia, neck stiffness, fever, dysphagia, respiratory, gastrointestinal symptoms or sick contacts. The patient denied any history of trauma to the neck or spine. Thirteen days before the onset of symptoms, the patient received his first dose of Oxford-AstraZeneca SARS CoV-2 Vaccine. 

A lumbar puncture was done and his CSF analysis revealed a markedly elevated protein at 186mg/dl, normal glucose at 73mg/dl and WBC at 0/mm^3^. This indicated an albuminocytological dissociation. CSF Gram stain and meningoencephalitis panel were both negative. His complete blood count, renal function test and liver function test were all within the normal range. The neurophysiological studies also revealed diffuse demyelination consistent with the acute inflammatory demyelinating polyneuropathy subtype. Based on the diagnostic parameters present in this case, Brighton criteria for level 4 were met thus confirming a diagnosis of Guillain-Barre syndrome. 

Further investigations revealed a normal CT Brain, CT Aortogram and MRI Cervical Spine (1.5T). Treatment was initiated with intravenous immunoglobulin (IVIG) at 400mg/kg/day for five days as well as aggressive physical therapy and gabapentin 300mg three times daily for pain control. This led to significant improvement in symptoms and he was discharged with partial resolution of his facial weakness but with significant improvement in his power making his GBS disability score 2. Upon clinical review in the outpatient setting two weeks later, there was no motor deficit apart from 4+/5 proximally bilaterally with minimal left facial weakness and total resolution of his pain. His GBS disability score improved to 1. 

Case 4: Meningitis retention syndrome associated with ChAdOx1 nCoV-19 vaccine

A 61-year-old female of South-Asian descent presented to the hospital with headache, fever, paresthesias of the calves and thighs bilaterally, diplopia, urinary retention, and ataxia. Symptoms began three days prior with fever, headaches, paresthesias and an unsteady gait. The following day, she developed diplopia and urinary retention. Her gait became progressively unsteady until the time of presentation. The patient received her first dose of Oxford-AstraZeneca SARS CoV-2 vaccine 18 days prior to the onset of symptoms. She denied photophobia, nausea, and vomiting. Her past medical history was significant for verrucous hyperplasia, successfully treated with surgery and selective radiotherapy three years previously. A Foley’s catheter was inserted and 1400ml of urine drained, which led to admission.

On clinical examination, she was alert and fully oriented with normal vital signs and a temperature of 36.1 degrees Celsius. Neck stiffness was noted, but Brudzinski and Kernig signs were absent. Fundoscopy revealed no papilledema, whilst the remainder of the ophthalmological examination revealed diplopia to extreme lateral gaze bilaterally. There were no lateralizing neurological signs, such as weakness or spasticity. Her gait was ataxic, but sensory examination was otherwise normal.

Her complete blood count, renal function test, liver function test and C-reactive protein were all within the normal range. MRI Brain with contrast, MRI cervical spine with contrast, nerve conduction study, electromyogram and whole-body CT scan were all performed. MRI brain revealed non-enhancing, non-specific deep white matter lesions but all other investigations were normal. Blood and urine cultures were negative for bacterial growth. Urodynamic testing was unavailable.

A lumbar puncture was performed which revealed clear, colorless CSF and analysis showed 200 white blood cells 70mm3 with lymphocytic predominance, a mildly elevated protein (65mg/dl, reference 12-60mg/dl) and glucose CSF to serum ratio of 0.5. CSF Gram stain and meningoencephalitis panel (polymerase chain reaction [PCR] for herpes simplex virus 1 & 2, human herpes virus 6, human parechovirus, varicella-zoster virus, cytomegalovirus, Escherichia coli, Haemophilus influenza, Listeria monocytogenes, Neisseria meningitides, Streptococcus pneumoniae, Streptococcus agalactiae and Cryptococcus neoformans/gattii) were both negative. Paraneoplastic antibodies (anti-Yo, anti-Hu, anti-Ri) and COVID-19 PCR swab were both negative.

Dexamethasone was commenced (10mg IV loading dose, then 4mg IV three times daily for three days, followed by 2mg IV three times daily for three days. The patient’s headache, fever and diplopia resolved four days later. The following day, her catheter was discontinued, and she was able to void without assistance. Ataxia progressively improved and there was only minor impairment on tandem gait at clinical review, two weeks after presenting to the hospital. The patient was reassessed at two months in the outpatient setting which revealed complete resolution of her ataxia and diplopia. There were some mild symptoms of urinary hesitancy that persisted.

After reviewing her clinical course as well as her extensive laboratory workup and cerebrospinal fluid analysis, her diagnosis was that of aseptic meningitis with urinary retention.

## Discussion

Several commonly used vaccines are associated with post-vaccination neurological complications. Vaccines such as those used for influenza, hepatitis A or B, human papillomavirus, rabies, measles, rubella, yellow fever, and tetanus are temporally associated with neurological adverse events. These include optic neuritis, transverse myelitis, encephalitis, and Guillain-Barre syndrome [6,7]. 

Vaccines for COVID-19 are not exempted from such adverse effects. Neurological symptoms reported after vaccination are commonly mild, transient symptoms such as headaches, dizziness, myalgia and paresthesias [8]. Garg and Paliwal [8] detailed the variety of neurological complications associated with COVID-19 vaccines by conducting a review of published articles. Their review detailed published reports of neurovascular complications such as cerebral venous sinus thrombosis, neuroinflammatory complications such as acute disseminated encephalomyelitis (ADEM), cranial nerve palsies such as Bell’s palsy, peripheral nerve complications such as Guillain-Barre syndrome, and neuromuscular complications such as myositis [8]. 

SARS-CoV-2 and adverse reactions to SARS-CoV-2 vaccines exhibit a tropism for neuronal structures and tissues [9]. Molecular mimicry has been proposed as a pathogenic mechanism for COVID-19 vaccine-related neurological side effects [10,11]. The spike proteins, which these vaccines produce antibodies against, can also bind to sialic acid-containing glycoproteins and gangliosides on cell surfaces [11]. This adds to the biological plausibility of these adverse events being due to the vaccine. The background incidence rates of neurological conditions in the population and the large numbers of persons being vaccinated means that some of these conditions will appear in the post-vaccination window by chance. Therefore, establishing a causal relationship between the vaccination and the neurological condition is difficult. This understanding is important as the adverse effects of vaccines are reported as the leading cause of vaccine hesitancy [12]. The close temporal relationship between vaccination and the presenting symptoms, the biological plausibility, and extensive diagnostic workup to exclude other common causes increase the likelihood that these adverse events were due to the vaccines. These factors fulfill some of the criteria provided by the World Health Organization for causality assessment of an adverse event following immunization on an individual level. However, establishing a causal link on a population level requires large epidemiological studies and cannot be done through individual case reports alone [13].

Currently, the four major vaccines being used are COVID-19 mRNA-based vaccines, viral vector-based vaccines, inactivated/attenuated vaccines, and protein-based vaccines. This case report examines four cases determined to be adverse events likely due to COVID-19 vaccines which are the following: one case of new-onset seizures following the Pfizer-BioNTech vaccine, one case of longitudinally extensive transverse myelitis following the Pfizer-BioNTech vaccine, one case of Guillain-Barre syndrome following the ChAdOx1 nCoV-19 vaccine and one case of a meningitis-retention syndrome following the ChAdOx1 nCoV-19 vaccine. These cases give an idea of the spectrum of neurological adverse events related to COVID-19 vaccines by covering diseases of the cerebrum, spinal cord, and peripheral nerves. 

Encephalitis, inflammation of the brain, is most commonly caused by viral infections (especially HSV-1) followed by autoimmune encephalitides, inclusive of anti-glutamic acid decarboxylase (GAD) antibody type. Anti-GAD encephalitis is an important consideration in this patient (Case 1) with new-onset insulin-dependent diabetes mellitus and new-onset seizures. GAD is an intracellular enzyme that converts glutamate to gamma aminobutyric acid (GABA) [14]. GABA acts as an inhibitory neurotransmitter in the central nervous system however the high levels in pancreatic islet cells are associated with paracrine signaling and GAD autoantibodies potentially developing with new-onset insulin-dependent diabetes mellitus [15]. High serum levels of anti-GAD antibodies as well as intrathecal synthesis of anti-GAD antibodies suggest the diagnosis of anti-GAD encephalitis. Anti-GAD encephalitis is typically poorly responsive to antiepileptic drugs [16]. There are no pathognomonic radiological findings for anti-GAD encephalitis and the EEG of anti-GAD-associated epilepsy is non-specific [17]. Anti-GAD antibody testing was not available in this setting. This patient’s condition was determined to be an adverse event likely due to the Pfizer-BioNTech vaccine given the close temporal relationship between vaccination and the presentation of their symptoms, in addition to the diagnostic workup to exclude other causes.

Patients with encephalitis typically present with changes of consciousness levels, seizures, fever, focal neurological deficits, and movement disorders [18]. Zuhorn et al. [19] detailed three cases of encephalitis following vaccination with ChAdOx1 nCov-19 vaccine. The onset of symptoms of encephalitis occurred within seven to 11 days of vaccination with the ChAdOx1 nCov-19 (AZD1222). From their analysis of publicly available databases, 79 cases of unexplained encephalitis occurred following 99.3 million doses of the ChAdOx1 nCov-19 (AZD1222) vaccine with a resulting incidence of almost 0.08 per 100,000. Twenty cases of unexplained encephalitis occurred following 110.6 million doses of the Pfizer-BioNTech mRNA vaccine (BNT162b2) with a resulting incidence of almost 0.02 per 100,000 [19]. The significant difference in case rates following the ChAdOx1 nCov-19 vaccine and Pfizer-BioNTech vaccine and lack of reports following other COVID-19 vaccines suggest a causal relationship [19]. Proof of a causal relationship will require large observational epidemiological studies that demonstrate a higher incidence of encephalitis following vaccination compared to the spontaneous occurrence of encephalitis following vaccination [19].

Acute transverse myelitis (ATM) is a focal inflammatory disorder of the spinal cord, resulting in motor, sensory, and autonomic dysfunction [20]. Transverse myelitis can occur as a parainfectious complication. It can also occur due to systemic inflammatory diseases or multifocal central nervous system diseases including multiple sclerosis (MS) and neuromyelitis optica spectrum disorder [21]. The annual incidence of ATM ranges from 1.34 to 4.60 cases per million, but increases to 24.6 cases per million if acquired demyelinating diseases like MS are included [21]. As of March 2021, nine cases of transverse myelitis were reported to the Vaccine Adverse Events Reporting System (VAERS) of the CDC in relation to COVID-19 vaccines used in the United States of America [4]. This was following the administration of 51,755,447 dosages of the COVID-19 vaccines [4]. Finsterer [9] in his review paper published in November 2021, reported 11 published cases of transverse myelitis following COVID-19 vaccines, listing this complication as the fourth most common neurological serious adverse effect. Seven cases occurred following the ChAdOx1 nCoV-19 vaccine while one published case occurred after an mRNA vaccine (Moderna) [9]. Fitzsimmons and Nance [22] described a case in which a 63-year-old male developed symptoms of transverse myelitis one day after the second dose of an mRNA vaccine (Moderna). 

In an interim analysis of four randomized controlled trials in Brazil, South Africa and the United Kingdom, three participants out of 11,636 participating subjects developed ATM, triggering a study pause for careful review. One participant developed ATM 14 days after a booster of the ChAdOx1 nCov-19 vaccine. The ATM experienced by the second participant was considered unlikely due to the vaccine after further investigations revealed underlying previous multiple sclerosis. The third participant, who developed ATM 68 days after vaccination, was a control subject who received the meningococcal conjugate vaccine. The study concluded that the vaccine was safe and efficacious [23].

The most common form of Guillain-Barre syndrome (GBS) is an acute inflammatory demyelinating polyradiculoneuropathy presenting as progressive motor weakness typically beginning in the legs and advancing proximally. The estimated annual incidence in the United States is up to 1.79 per 100,000 persons [24]. Molecular mimicry is a proposed pathogenic mechanism for GBS following vaccination with COVID-19 vaccination. An antibody cross reaction between the spike protein target and cell surface molecules (sialic acid-containing glycoproteins and gangliosides) may be the causal link between GBS and immunization to SARS-CoV-2 [11]. Thirty-two cases of GBS following vaccination have been reported to the VAERS of the CDC as of March 2021 [4]. Finsterer [9] reports 389 patients with GBS following COVID-19 vaccination as of the end of September 2021, making this the second most common neurological adverse event. However, in their September 2021 statement, the Global Medical Advisory Board of the GBS/Chronic Inflammatory Demyelinating Polyneuropathy Foundation stated that they have not seen evidence that COVID-19 vaccines are more likely to cause GBS even in persons who have had GBS brought on due to other vaccines [25].

The combination of aseptic meningitis (AM) and acute urinary retention has been termed meningitis-retention syndrome (MRS) [26]. Typical clinical manifestations of MRS include fever, headache, neck stiffness, nausea/vomiting, minor pyramidal signs and acute urinary retention [27,28].

The majority of published cases of MRS display CSF findings consistent with those reported here: mononuclear pleocytosis, elevated protein and a decreased glucose CSF:serum ratio [27]. Urinary retention in MRS is believed to be of neurologic etiology, the exact mechanism of which is uncertain [27,28]. Several theories have been proposed; most commonly spinal shock secondary to meningeal irritation and an underlying mild acute disseminated encephalomyelitis (ADEM) [27,29]. The initial belief [27,29] that MRS was a mild form of ADEM was later deemed unlikely in light of new reports of MRS not restricted to aseptic meningitis [28]. Urodynamic studies have confirmed hypotonic or atonic neurogenic bladder in several cases of MRS [27,29,30]. Detrusor areflexia was common to all cases, resulting in an inability to achieve intravesical pressures sufficient to initiate micturition [27,29,30]. Urinary retention typically resolves within two weeks, after complete recovery from meningitis symptoms [27-30]. This resolution without immune treatment further casts doubt on the theory that MRS may be a mild form of ADEM [28].

Ataxia is infrequently reported in combination with urinary retention and aseptic meningitis/meningoencephalitis. A reversible splenial lesion in the corpus callosum was observed radiologically in these cases [31-33]. Although the patient discussed here exhibited similar signs and symptoms to those patients, such as headache, fever, urinary retention and gait ataxia, our case differed in that we observed neither splenial lesions nor myelitis on cranial and spinal MRI respectively. Ataxia and urinary retention are common to mild encephalitis/encephalopathy with a reversible splenial lesion (MERS) and ADEM and further studies are required to evaluate whether these diseases and MRS may represent a spectrum of the same disease [32,33]. Ataxia has been previously reported to occur within six weeks of vaccination, and it has been suggested that some of these may indeed have been triggered by the administered vaccine [34].

The MRS experienced by the patient in Case 4 was determined to be an adverse effect likely due to the ChAdOx1 nCoV-19 vaccine, considering the close temporal association between the administration of the vaccine and the diagnostic workup which excluded infectious meningitis and other causes of AM such as systemic disease, drugs, solid cancer metastasis and malignant hemopathy. This is, to the best of our knowledge, the first reported case of vaccine-induced MRS, and the first case of MRS accompanied by ataxia without an observable splenial lesion.

## Conclusions

Cases of neurological diseases affecting the various components of the central and peripheral nervous system following COVID-19 vaccinations have been reported. The background incidence rates of these neurological diseases and the large number of persons being vaccinated means that some of these conditions will occur in the post-vaccination window by chance. Hence, establishing a causal link on a population level requires large epidemiological studies and cannot be done on individual case reports alone. The close temporal relationship between vaccination and the presenting symptoms, the biological plausibility, and the extensive diagnostic workup to exclude other causes fulfill criteria provided by the World Health Organization for causality assessment of an adverse event following immunization on an individual level. On this basis, it was determined that these adverse events were likely due to the vaccines. While these adverse events appear to be rare, physicians should still be cognizant of them.
